# Cases of tetrasomy 9p and trisomy 9p in prenatal diagnosis—Analysis of noninvasive and invasive test results

**DOI:** 10.3389/fgene.2022.994455

**Published:** 2022-09-26

**Authors:** Hanna Moczulska, Michal Pietrusinski, Karolina Zezawska, Marcin Serafin, Beata Skoczylas, Tomasz Jachymski, Katarzyna Wojda, Piotr Sieroszewski, Maciej Borowiec

**Affiliations:** ^1^ Department of Clinical Genetics, Medical University of Lodz, Lodz, Poland; ^2^ Department of Fetal Medicine and Gynecology, Medical University of Lodz, Lodz, Poland

**Keywords:** tetrasomy 9p, trisomy 9p, prenatal diagnosis, amniocentesis, microarrary

## Abstract

**Objective:** Tetrasomy 9p and trisomy 9p are rare chromosomal aberrations. The phenotypes of tetrasomy 9p and trisomy 9p are variable. Most cases are diagnosed in the postnatal period. The study aims to analyze the prenatal phenotype of tetrasomy 9p and trisomy 9p in terms of ultrasound and screening tests.

**Methods:** A set of 1573 prenatal tests performed from 2016 to 2021 was reviewed to identify all cases with trisomy 9p and tetrasomy 9p. In four cases with 9p gain, non-invasive and invasive test results were analyzed.

**Results:** Four cases with the 9p gain were diagnosed in the prenatal period: two cases with tetrasomy 9p and two cases with trisomy 9p. Nasal bone hypoplasia and ventriculomegaly are common features of 9p gain. In two out of four cases with the 9p gain, an increased risk of trisomy 21 was found in the combined first-trimester screening test.

**Conclusion:** Trisomy 9p and tetrasomy 9p are characterized by a variable phenotype in the prenatal period, manifesting in genetically abnormal fetuses. The tetrasomy 9p and trisomy 9p may suggest trisomy 21 in the first trimester.

## Introduction

Tetrasomy 9p is a rare chromosomal aberration caused by the presence of four copies of the short arm of chromosome 9. The extra two copies form an additional isochromosome. Most cases of tetrasomy 9p occur *de novo* during maternal meiosis II nondisjunction (Dutly et al., 1998). The short arm is duplicated and the long arm is lost. About 60 cases of tetrasomy 9p have been published, including over 20 prenatal cases (Vinkšel et al., 2019). Individuals with tetrasomy 9p demonstrated brain, heart, and genitourinary system malformations. Additionally, facial dysmorphia and intellectual disability were observed in patients with tetrasomy 9p. Fewer and less severe abnormalities were observed in cases with mosaicism (Chen et al., 2014). Symptoms vary in affected individuals, depending on the type and percentage of cells containing tetrasomy 9p. A prenatal diagnosis of mosaic tetrasomy 9p is rare. A false negative result in pregnancies with fetal mosaic tetrasomy 9p may be obtained, because the mosaic level of tetrasomy 9p may decrease after long-term tissue culture (Chen et al., 2014).

The phenotype of trisomy 9p is milder. More than 150 cases of trisomy 9p have been described. Most cases with trisomy 9p demonstrate a parental reciprocal translocation between chromosome nine and another autosome. In 9p trisomy, duplication may involve part of the short arm, the whole short arm, or the short arm, and part of the long arm of chromosome 9. The symptoms of 9p trisomy may be similar in affected individuals, regardless of the size of the duplicate 9p part. Trisomy 9p is usually diagnosed in the postnatal period (Guilherme et al., 2014). Little information has been published about the fetal phenotype of trisomy 9p; however, it is likely that trisomy 9 has a milder phenotype that does not allow detection during pregnancy.

As gains in 9p can be diagnosed invasively with karyotyping or aCGH and not by routine screening tests, the aim of our study was to analyze the prenatal phenotype of cases with trisomy 9p and tetrasomy 9p in ultrasound and screening tests.

## Materials and methods

A sample of 1573 prenatal tests (1409 amniocentesis and 164 chorionic villus sampling) performed in the Department of Clinical Genetics of the Medical University of Lodz from 2016 to 2021 were reviewed to identify cases with trisomy 9p and tetrasomy 9p. The search identified four cases with 9p gain; in these cases, non-invasive and invasive test results were analyzed. In addition, a review of the literature was performed.

**FIGURE 1:**
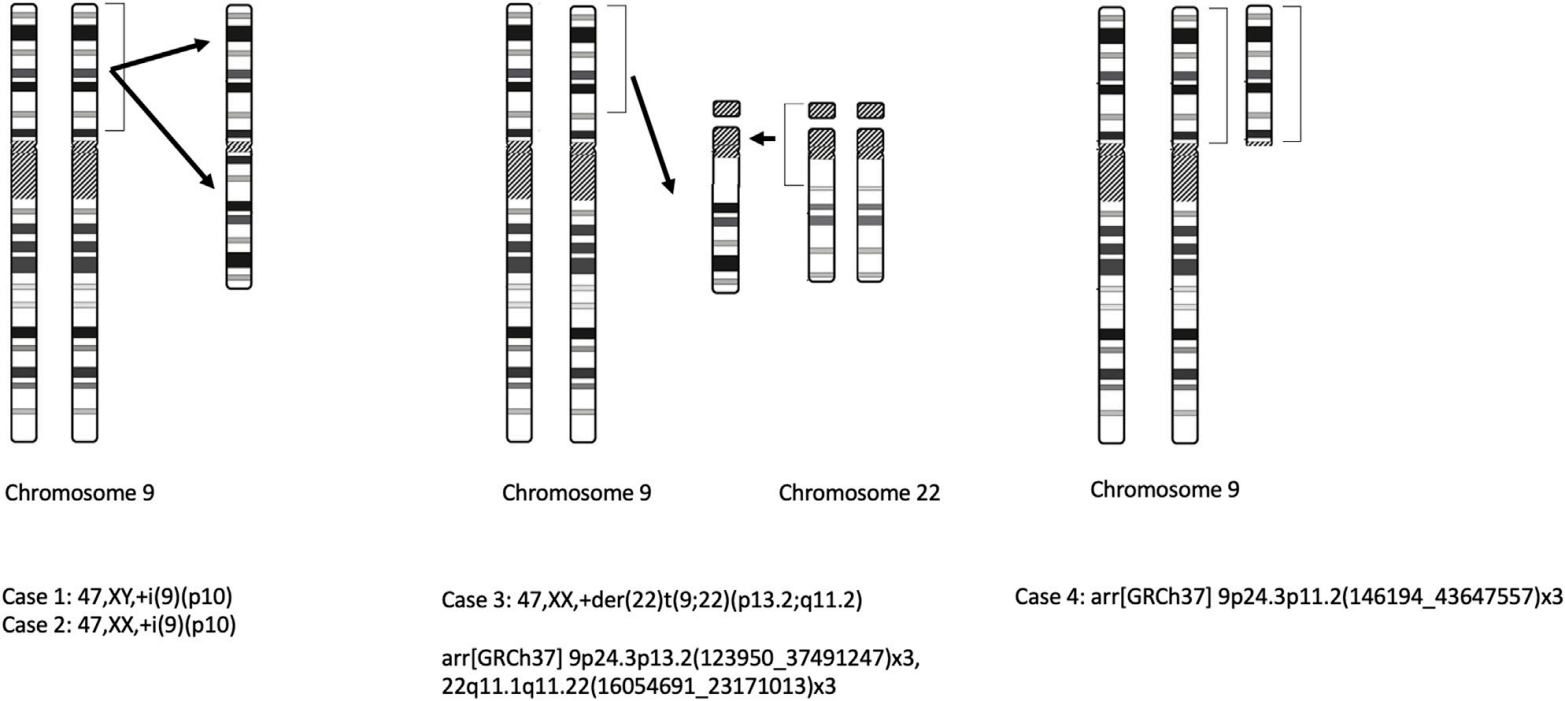
Schematic drawings of the observed chromosomal aberrations (case 1, 2, 3, 4).

All prenatal genetic tests were ordered in response to a diagnosis of high-risk pregnancy in the Department of Clinical Genetics and the Department of Fetal Medicine and Gynecology of the Medical University of Lodz. The study itself was conducted in a tertiary referral center. The study group comprised women at high risk of having a child with a genetic disease. All patients were Caucasian. The patients were tested in the National Health System’s prenatal screening program. The criteria for inclusion in the prenatal testing program comprises any of the following: the age above 35 years, the occurrence of a chromosomal aberration in the previous fetus or child, structural chromosomal aberration in the mother or father of the fetus, a significantly increased risk of having a child with a monogenic or multifactorial disease, abnormal fetal ultrasound or biochemical tests results that indicate increased the risk of chromosomal abnormalities or fetal abnormalities. Detailed ultrasound examinations were performed in all cases. In most cases, fetal echocardiography was performed, especially in cases of suspected fetal heart defects, in fetuses with non-cardiac defects, fetal chromosomal abnormalities, and in cases with a family history of heart defects. All scans were carried out by certified sonographers (certificates of the Foetal Medicine Foundation and the Polish Society of Gynaecologists and Obstetricians). Non-invasive tests consisted of the combined test (reimbursed in the National Health System’s prenatal screening program) and the non-invasive prenatal screening testing NIPT (not reimbursed in Poland). Invasive procedures were indicated by abnormal fetal ultrasound, high risk of aneuploidy in screening tests, or history indicating an increased risk of genetic disease in the fetus. Fetal material was collected by amniocentesis or chorionic villus sampling. In each case, the clinical geneticist selected the appropriate genetic test. In our department, the first-line test is aCGH (array-based comparative genomic hybridization). In selected cases, the diagnosis is completed by an assessment of karyotype or molecular tests. aCGH was performed using Agilent, GenetiSure Pre-Screen Kit 8x60K with a resolution of approximately 0.50 Mb. According to standard protocols, samples were cultured for classical cytogenetic analysis. The chromosomes obtained in the metaphase were subjected to Giemsa staining after trypsin treatment and analyzed on the Cytovision karyotyping platform (Cytovision DM2500) (Caspersson et al., 1968; Sumner, 1982). Karyotype and aCGH results are described according to the International System for Human Cytogenomic Nomenclature (ISCN 2020) (Liehr, 2021). Schematic drawings of the observed chromosomal aberrations for all 4 cases are demonstrated in Figure 1. The study was conducted according to the Declaration of Helsinki on human subject research. The participants gave their written informed consent authorizing us to perform genetic tests and use the data for research and education.

## Results

Four cases of 9p gain were diagnosed in the prenatal period: two cases with tetrasomy 9p and two cases with trisomy 9p (one case had an additional 22q11 duplication). Table 1 contains the prenatal sonographic features for the four described cases and other cases from the literature.

**TABLE 1:** Prenatal sonographic features of four described cases and cases from the literature.

		First trimester	Second and third trimester									
			Ventriculo megaly	DWM	CCA	CLP	NB hypoplasia	Micro gnathia	Cardiac anomaly	Limb	GU anomaly	FGR
										Deformation		
1	Case 1—tetrasomy 9p	↑ NT, NB(-)	x	x		x	x		x	x	x	x
2	Case 2—tetrasomy 9p	NA	x	x	x	x	x	x				
3	Case 3—trisomy 9p + dup 22q11	NB(-)	x	x			x		x	x	x	
4	Case 4—trisomy 9p	normal NT, NB	x							x		
5	Cazorla Calleja et al. Cazorla Calleja et al., (2003)—tetrasomy 9p	NA	x									x
6	Deurloo et al. Deurloo et al., 2004—tetrasomy 9p	NA	x	x							x	
7	Dhandha et al. Dhandha et al., 2002 case 1—tetrasomy 9p	NA	x	x		x						x
8	Dhandha et al. (Dhandha et al., 2002) case 2—tetrasomy 9p	NA				x			x	x	x	x
9	Dhandha et al. Dhandha et al., 2002 case 3—tetrasomy 9p	NA		x							x	x
10	Di Vera et al. di Vera et al., 2008—tetrasomy 9p	↑ NT	x	x		x			x		x	x
11	Dutly et al. Dutly et al., 1998 case 1– tetrasomy 9p	NA				x						x
12	Dutly et al. Dutly et al., 1998 case 2– tetrasomy 9p	NA								x		x
13	Hengstschlager et al. Hengstschläger et al., 2004—tetrasomy 9p	NA		x					x	x		
14	Jalal et al. Jalal et al., 1991—tetrasomy 9p	NA	x			x				x	x	
15	Khattabi et al. Zaghi et al., 2022 case 1—tetrasomy 9p	↑ NT, NB(-), CLP										
16	Khattabi et al. Khattabi et al., 2015 case 2—tetrasomy 9p	NA	x	x	x						x	x
17	Khattabi et al. Khattabi et al., 2015 case 3—tetrasomy 9p	↑ NT										
18	Kok Kilic et al. Kok Kilic et al., 2022—tetrasomy 9p	↑ NT	x			x		x	x			
19	Lazebnik et al. Lazebnik et al., 2015—tetrasomy 9p	normal NT, NB		x	x			x		x		x
20	McDowall et al. McDowall et al., 1989—tetrasomy 9p	NA	x	x	x	x						
21	Nakamura-Pereira et al. Nakamura-Pereira et al., 2009—tetrasomy 9p	↑ NT	x	x		x		x		x	x	
22	Podolsky et al. Podolsky et al., 2011—tetrasomy 9p	↑ NT, NB(-)							x			
23	Schaefer et al. Schaefer et al., (1991)—tetrasomy 9p	NA	x								x	
24	Tan et al. Tan et al., 2007—tetrasomy 9p	NA				x				x		x
25	Tang et al. Tang et al., (2004)—tetrasomy 9p	NA	x			x				x	x	
26	Vinksel et al. Vinkšel et al., 2019—tetrasomy 9p	↑ NT				x					x	
27	Wang et al. Wang et al., 2015—tetrasomy 9p	NA							x			

NT, nuchal translucency; NB, nasal bone; TR, tricuspid regurgitation; DWM, Dandy-Walker malformation; CCA, corpus callosum agenesis; GU, genitourinary; FGR, foetal growth restriction; CLP, cleft lip and palate; NA, not available

### Case 1

A 32-year-old gravida, G3P1A1 (gravida, para, abortus), underwent amniocentesis at 16 weeks of gestation because of a high risk of fetal trisomy 21. There was no family history of congenital malformations. The nuchal translucency (NT) measured 3.0mm, and the nasal bone was absent. The combined first-trimester screening revealed a high risk for Down syndrome 1:35 [PAPP-A (pregnancy-associated plasma protein-A) 0.57 MoM (multiple of the median); BhCG (the β-subunit of human chorionic gonadotropin) 0.46 MoM]. NIPT (non-invasive prenatal testing) indicated a low risk of trisomy 21, 18, and 13. Amniocentesis was performed at 16 weeks of gestation. Fetal aCGH demonstrated 9p24.3p13.1 gain: arr [GRCh37] 9p24.3p11.2(146194_43647557)x3. Fetal karyotype identified a complete tetrasomy of the short arm of chromosome 9 47,XY,+i(9)(p10) (Liehr, 2021). Second-trimester sonography (19 weeks of gestation) showed nasal bone hypoplasia (4.4mm; <1^st^ percentile), cleft lip and palate, abnormal posterior fossa (Dandy-Walker malformation), ventriculomegaly (posterior ventricle Vp 10.3 mm), ventricular septal defect, and humerus and femur shortening (humerus length HL 11^th^ percentile; femur length FL 9^th^ percentile). The pregnancy was terminated. No postmortem examination was performed.

### Case 2

33-year-old gravida, G1P0A0, was consulted in the course of the first gestation. She was exposed to harmful medications for atopic dermatitis in early pregnancy—tetracycline and retinoid derivatives. The family and medical histories were unremarkable.

Medical data from the first trimester were unavailable, the patient reported that the ultrasound result was normal, and the combined test was not performed. Second-trimester sonography (18 weeks of gestation) demonstrated multiple abnormalities: agenesis of the corpus callosum, ventriculomegaly (Vp 11 mm), abnormal posterior fossa (Dandy-Walker malformation), bilateral cleft lip and palate, absent nasal bones, and retrognathia. In addition, pericardial effusion, increased renal echogenicity and pyelectasis, and clenched hands were found. Diagnostic amniocentesis was performed. Fetal karyotype showed following results—47,XX,+i(9)(p10). The pregnancy was terminated. No postmortem examination was performed.

### Case 3

A 32-year-old gravida, G2P2A1, underwent amniocentesis at 19 weeks of gestation because of the high risk of fetal trisomy 21 and multiple malformations. There was no family history of congenital disabilities. At 13 weeks of pregnancy, an ultrasound examination revealed absent nasal bone, tricuspid regurgitation, and bradycardia (fetal heart rate 134/min.). The combined first-trimester screening revealed a high risk for Down syndrome 1:47 (PAPP-A 0.88 MoM; BhCG 4.36 MoM). NIPT indicated a low risk of trisomy 21, 18, and 13. The scope of NIPT included risk assessment for trisomy 21, trisomy 18, trisomy 13 and sex chromosomal abnormalities. At that time, the patient decided against an invasive procedure. Second-trimester sonography demonstrated ventriculomegaly (Vp 10 mm), nasal bone hypoplasia, and ARSA (aberrant right subclavian artery). There was also suspicion of corpus callosum partial agenesis and Dandy-Walker malformation. At 19 weeks the patient underwent amniocentesis. After sampling the amniotic fluid, aCGH was performed. The following aberrations were revealed: arr[GRCh37] 9p24.3p13.2(123950_37491247)x3,22q11.1q11.22(16054691_23171013)x3. Fetal karyotype was not assessed due to cell culture failure. Sonography at 22 weeks showed Dandy-Walker malformation, mild ventriculomegaly (Vp 10 mm), hypoplasia of corpus callosum, nasal bones hypoplasia (3.9mm; <1^st^ percentile), and shortening of the humerus and femur (FL 35mm; 3^rd^ percentile, HL 31mm; 2^nd^ percentile). Moreover, tricuspid regurgitation and pulmonary stenosis (Vmax = 95 cm/s) were detected.

At 28 and 32 weeks, subsequent examination showed mild pulmonary stenosis (Vmax = 105 cm/s), previously reported abnormalities in the brain, mild renal pelvis dilatation (5.5 mm), nasal bone hypoplasia (5.3mm; <1^st^ percentile), long bones shortening (FL 35mm; 3^rd^ percentile, HL 44mm; 1^st^ percentile) and polyhydramnios (amniotic fluid index - AFI 30 cm). The baby was born in the 39th week of pregnancy, Apgar 9/9, weight 3000g and length 51 cm. The newborn karyotype revealed an additional derivative chromosome: 47,XX,+der (22)t (9;22) (p13.2;q11.2)dmat. The mother of the child was a carrier of a balanced translocation: 46,XX,t(9;22)(p13.2;q11.2). The father’s karyotype was normal: 46,XY.

### Case 4

A 28-year-old, G3P1A1, was referred to the clinic for suspected congenital fetal defects in second-trimester sonography. First-trimester sonography was normal. The combined first-trimester screening revealed a low risk for trisomy 21, 18, and 13 (PAPP-A 0.43 MoM; BhCG 1.19 MoM); risk for trisomy 21 1:1519, risk for trisomy 18 < 1:20000 and risk for trisomy 13 1:2275. At 21 weeks of gestation, bilateral ventriculomegaly (Vp 11.8 mm), club foot, and fetal growth restriction (10^th^ percentile) were detected. Amniocentesis was performed at 24 weeks of gestation. aCGH study demonstrated trisomy: arr[GRCh37] 9p24.3p11.2 (146194_43647557)x3 (43.5 Mbp). Negative test results for cytomegalovirus (CMV) and *Toxoplasma gondii* were obtained. Parents reported that the delivery was at 39 weeks, and genetic testing in the newborn confirmed prenatal diagnosis.

## Discussion

Our paper presents two prenatal cases of tetrasomy 9p and two cases of trisomy 9p. Tetrasomy 9p is characterized by a variable phenotype in the prenatal period (Dhandha et al., 2002; Vinkšel et al., 2019). The fetal phenotype of trisomy 9p is poorly described in the literature. Leichtman et al. described phenotypic overlap between tetrasomy 9p and trisomy 9p, and noted that tetrasomy 9p presents a more severe phenotype. The spectrum of symptoms may be continuous between tetrasomy 9p, mosaic tetrasomy 9p, and trisomy 9p. The phenotype may depend on the gene dosage effect (Leichtman et al., 1996). Our study indicates a similar relationship, with cases with tetrasomy 9p having a more severe phenotype (cases 1 and 2).

The clinical data of all the prenatal cases with tetrasomy 9p, plus our two cases with tetrasomy 9p and our two cases with 9p trisomy, are given in Table 1. Comparing cases from the literature is challenging, as they have been published in the last 10–20 years, and the quality of ultrasound examinations has increased significantly during this time. Nevertheless, the table shows that the most common defects in fetuses with tetrasomy 9p are ventriculomegaly, Dandy-Walker malformation, and cleft lip/palate (Table 1). These defects are quite unspecific and can be found in many genetically abnormal fetuses.

Brain defects are often reported in fetuses with tetrasomy 9p, with the most common abnormalities being ventriculomegaly and Dandy-Walker malformation. Deurloo et al. suggested that Dandy-Walker malformation may be a marker of tetrasomy 9p (Deurloo et al., 2004). In our study, all cases had ventriculomegaly, and three out of four had Dandy-Walker malformation. Agenesis of the corpus callosum is also observed in fetuses with tetrasomy 9p (McDowall et al., 1989; Lazebnik and Cohen, 2015). Two cases in our study demonstrated corpus callosum hypoplasia/agenesis (cases 2 and 3).

In cases of tetrasomy 9p, fetal facial dysmorphia consists of cleft lip and palate, micrognathia, and abnormal facial profile. Hypertelorism is often found in postnatal diagnosis (Jalal et al., 1991). Until now, binocular distance (BOD) has not been studied in prenatal cases with tetrasomy 9p, nor were the nasal bones. Podolsky et al. presented one case with the absence of nasal bone as a marker of tetrasomy 9p (Podolsky et al., 2011). Zaghi et al. described one case with tetrasomy 9p and absent nasal bone (Zaghi et al., 2022). Khattabi et al. presented a case with tetrasomy 9p and absent nasal bone diagnosed in the first trimester (Khattabi et al., 2015). In our study, three of the four fetuses had nasal bone hypoplasia. In two cases, nasal bone hypoplasia was diagnosed in the first trimester (cases 1 and 3).

Congenital heart defects are observed in 30% of fetuses with tetrasomy 9p, with the most common being defects in the ventricular septal and common atrioventricular canal, and complex heart defects (Dhandha et al., 2002; Kok Kilic et al., 2022). Interestingly, a persistent left superior vena cava is often seen, which may be the only symptom of tetrasomy 9p. Wang et al. presented a prenatal diagnosis of mosaic tetrasomy 9p in a fetus with isolated persistent left superior vena cava (Wang et al., 2015).

Defects of the genitourinary system are observed in 43% of cases with tetrasomy 9p. The severity of the lesions varies significantly from mild pyelectasia to bilateral multicystic dysplastic kidney. The amount of amniotic fluid is often abnormal and polyhydramnios is mainly observed (Tan et al., 2007). Oligohydramnios is found in fetuses with tetrasomy 9p and severe urinary tract defects (Schaefer et al., 1991).

The abnormalities caused by tetrasomy 9p were usually found in the second or third trimester. Single reports of tetrasomy 9p have been reported after first-trimester diagnosis (Nakamura-Pereira et al., 2009; Kok Kilic et al., 2022). Fortunately, modern ultrasound technology allows faster and more accurate early diagnosis of developmental abnormalities. In our group, nuchal translucency was increased in one case with 9p gain, and nasal bone hypoplasia was observed in three cases: two cases were diagnosed in the first trimester. A combined first-trimester screening test indicated a high risk of trisomy 21 in two fetuses (case 1, case 3). Our findings suggest that the clinical picture of the 9p gain in the first trimester may suggest the most common trisomies. Therefore invasive testing should be recommended in cases with a high risk for trisomy 21 (>1:100) and normal NIPT, including aCGH. Common features include increased NT, absence of nasal bones, tricuspid regurgitation, and abnormal first-trimester maternal serum screening test. Our cases demonstrated decreased levels of PAPP-A (0.57 MoM, 0,88 MoM and 0.43 MoM) and variable free B-hCG levels (0.46 MoM, 4.3 MoM and 1.19 MoM). Khattabi et al. described a case with tetrasomy 9p in which maternal serum screening indicated a high risk of trisomy 21 (Khattabi et al., 2015); however, Lazebnik et al. presented a prenatal case with tetrasomy 9p with a low risk for trisomy 21, 18, and 13 (serum analytes: 1.11 MoM PAPP-A and 1.75 MoM BhCG) (Lazebnik and Cohen, 2015). The relationship between first-trimester maternal screening and the fetal 9p gain has not been investigated; as such, more cases are needed to thoroughly analyze the relationship between PAPP-A and BhCG levels and the fetal 9p gain.

Non-invasive prenatal testing (NIPT) is not routinely used in the prenatal screening of trisomy 9p and tetrasomy 9p, and hence few publications discuss this possibility. Wang et al. presented a case with mosaic tetrasomy 9p in the fetus, in which NIPT revealed “extra” genetic material derived from chromosome 9 (Wang et al., 2015). The first case of maternal mosaic tetrasomy 9p being incidentally detected on NIPT was reported by Shu et al.; in this case, NIPT was performed twice and both results revealed multiple chromosomal aberrations including elevation in DNA from chromosome 9p. The scope of NIPT included risk assessment for trisomy 21, trisomy 18, trisomy 13, sex chromosomal abnormalities, and genome-wide chromosomal aberrations at a resolution of 3 Mb or above. Amniocentesis was performed and a normal fetal aCGH result was obtained. Maternal blood karyotype revealed mos 47,XX,+dic(9;9)(q21.1;q21.1)[24]/46,XX[9], maternal fibroblasts were not examined. Subtelomeric multiplex ligation-dependent probe amplification (MLPA) was performed on uncultured maternal blood and on the maternal buccal swab. The blood test confirms mosaic 9p duplication, and the buccal swab result was normal. As the amniocentesis was normal, no clinical description or genetic testing was performed on the child after delivery (Shu et al., 2021). This case confirms that some cases with mosaic tetrasomy 9p may go undiagnosed in the general population.

In our study, the karyotype of case 3 shows the presence of derivative chromosome 22: 47,XX,+der(22)t(9;22)(p13.2;q11.2)dmat. 22q11.2 duplication syndrome is a condition characterized by variable clinical phenotype that includes heart defects, urogenital abnormalities, velopharyngeal insufficiency with or without cleft palate, mild learning difficulties with some individuals being essentially normal. The duplicated region contains 30 to 40 genes, however for many of these genes, little is known about their function. Genes that may influence the phenotype may be similar to genes involved in the same deleted region in DiGeorge syndrome (DGS; OMIM 188400) and velocardiofacial syndrome (VCFS; OMIM 192430) (mainly *TBX1*, *HIRA*, *CRKL*).

Trisomy 9p and tetrasomy 9p can be diagnosed using classical cytogenetic analysis and aCGH. The karyotype identifies the structure of a chromosomal aberration. aCGH offers higher test resolution than traditional G-band karyotyping and allows for more precise localization of the chromosomal breakpoints. Nowadays whole exome sequencing (WES) is sometimes performed as a first-line genetic test. WES can also diagnose chromosomal aberrations; however, it is not a good tool to diagnose them. The biggest advantage of microarrays over WES methods is the quality of the data post-processing in detecting specific copy number variations (CNVs). This is because WES methods mimic a traditional microarray method when so-called “pseudo probes” are created from the next-generation sequencing (NGS) reads to establish a log2 ratio value, which can then be used to estimate the actual copy number. In contrast, microarrays have “real probes” and during experiments, the signal intensity of these probes is compared directly to reference probes. Thus microarrays offer better overall genomic coverage and can detect intronic and intergenic alterations.

## Conclusion

Trisomy 9p and tetrasomy 9p are characterized by a variable phenotype in the prenatal period which can be found in many genetically abnormal fetuses. The clinical picture of the 9p gain in the first trimester may suggest trisomy 21.

## Data Availability

The raw data supporting the conclusion of this article will be made available by the authors, without undue reservation.
